# Vascular organoids: unveiling advantages, applications, challenges, and disease modelling strategies

**DOI:** 10.1186/s13287-023-03521-2

**Published:** 2023-10-10

**Authors:** Hojjat Naderi-Meshkin, Victoria A. Cornelius, Magdalini Eleftheriadou, Koray Niels Potel, Wiwit Ananda Wahyu Setyaningsih, Andriana Margariti

**Affiliations:** 1 The Wellcome-Wolfson Institute for Experimental Medicine, Queen’s University Belfast, 97 Lisburn Road, Belfast, BT9 7BL UK; 2 Department of Anatomy, Faculty of Medicine, Public Health, and Nursing, Universitas Gadjah Mada, Sleman, D.I. Yogyakarta 55281 Indonesia

**Keywords:** Vascular disease modelling, Vasculopathy, Angiopathy, Organ-specific endothelial dysfunction, Pluripotent stem cells, Microvascular and macrovascular complications

## Abstract

Understanding mechanisms and manifestations of cardiovascular risk factors, including diabetes, on vascular cells such as endothelial cells, pericytes, and vascular smooth muscle cells, remains elusive partly due to the lack of appropriate disease models. Therefore, here we explore different aspects for the development of advanced 3D in vitro disease models that recapitulate human blood vessel complications using patient-derived induced pluripotent stem cells, which retain the epigenetic, transcriptomic, and metabolic memory of their patient-of-origin. In this review, we highlight the superiority of 3D blood vessel organoids over conventional 2D cell culture systems for vascular research. We outline the key benefits of vascular organoids in both health and disease contexts and discuss the current challenges associated with organoid technology, providing potential solutions. Furthermore, we discuss the diverse applications of vascular organoids and emphasize the importance of incorporating all relevant cellular components in a 3D model to accurately recapitulate vascular pathophysiology. As a specific example, we present a comprehensive overview of diabetic vasculopathy, demonstrating how the interplay of different vascular cell types is critical for the successful modelling of complex disease processes in vitro. Finally, we propose a strategy for creating an organ-specific diabetic vasculopathy model, serving as a valuable template for modelling other types of vascular complications in cardiovascular diseases by incorporating disease-specific stressors and organotypic modifications.

**Graphical abstract:**

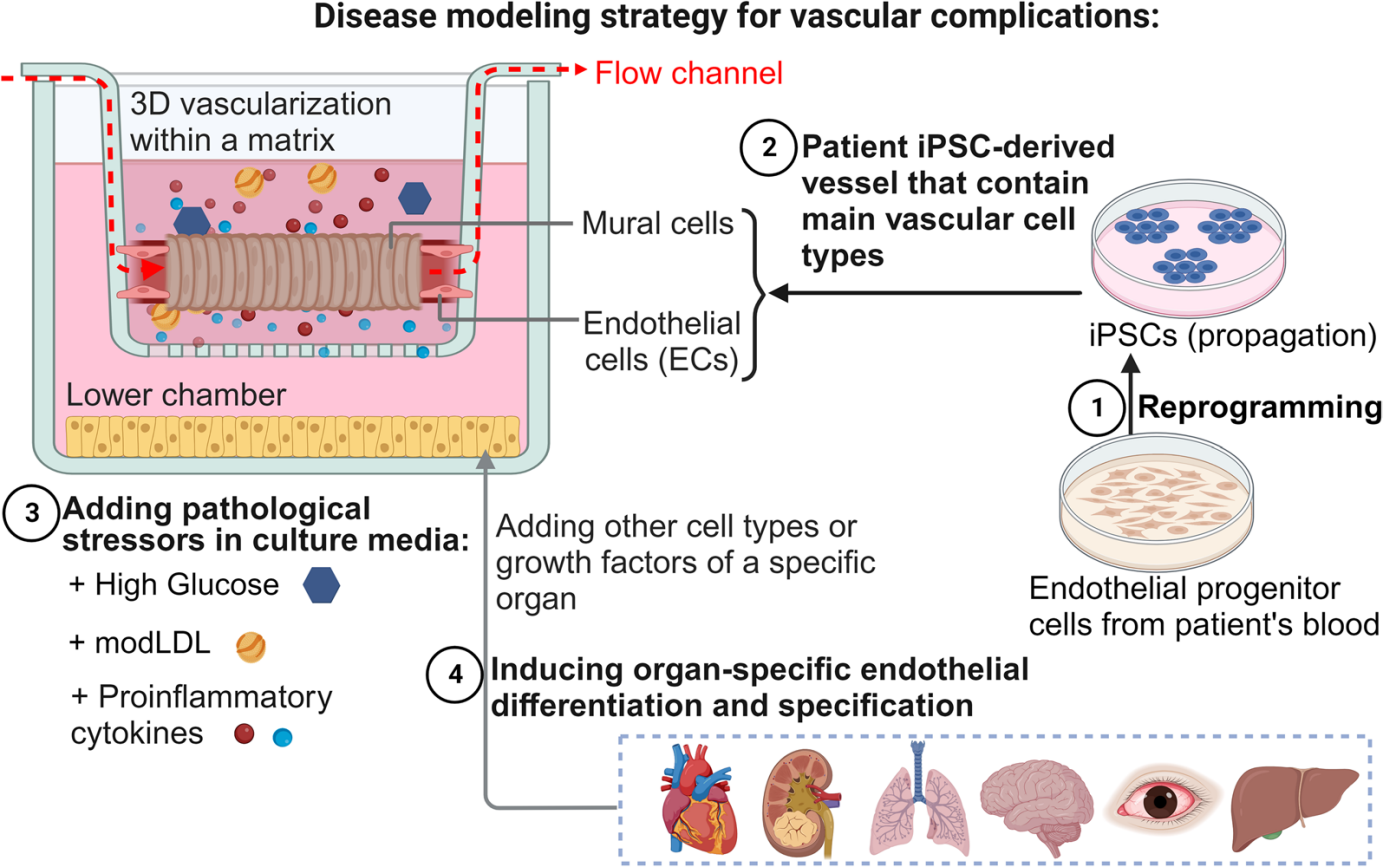

## Introduction

Vascular complications, encompassing cardiovascular diseases, atherosclerosis, diabetic retinopathy, and nephropathy, present formidable challenges to public health. Despite progress in diabetes management, these complications persist, profoundly affecting the well-being and life expectancy of individuals with diabetes [1]. Advancing our comprehension and management of these complexities demands enhanced disease models.

In recent years, in vitro disease models, particularly 3D vascular organoids (VOs), have emerged as valuable tools for studying diabetic vasculopathy/angiopathy and advancing our knowledge of microvascular and macrovascular complications [2, 3]. These organoids offer a more physiologically relevant platform compared to traditional animal models and 2D cell cultures. By mimicking the complex three-dimensional structure of in vivo organs, organoids provide insights into organ-specific functions and disease mechanisms [2, 4–6].

Human pluripotent stem cells (hPSCs) have played a pivotal role in the development of organoids. With their self-renewal capacity and ability to differentiate into various cell lineages, hPSCs offer an unlimited source of cells for generating organoids representing different organs, including VOs [4]. However, creating organoids that faithfully recapitulate the complexity of in vivo organs remains a challenge.

In this review, we explore the advancements in iPSC-derived 3D VOs, uncovering their advantages and applications. We also delve into the limitations encountered and discuss emerging solutions that aim to address these challenges. Drawing from our experiences in developing blood vessel organoids to model diabetic vasculopathy disease [2, 7], we present an in-depth analysis of the considerations and strategies involved in creating more physiologically relevant VOs.

Through this comprehensive review, we aim to shed light on the potential of iPSC-derived 3D VOs as transformative tools in understanding vascular complications and driving advancements in regenerative medicine and therapeutic approaches.

## Advantages of iPSC-derived 3D vascular organoids: overcoming limitations of 2D cultures

**Fig. 1:**
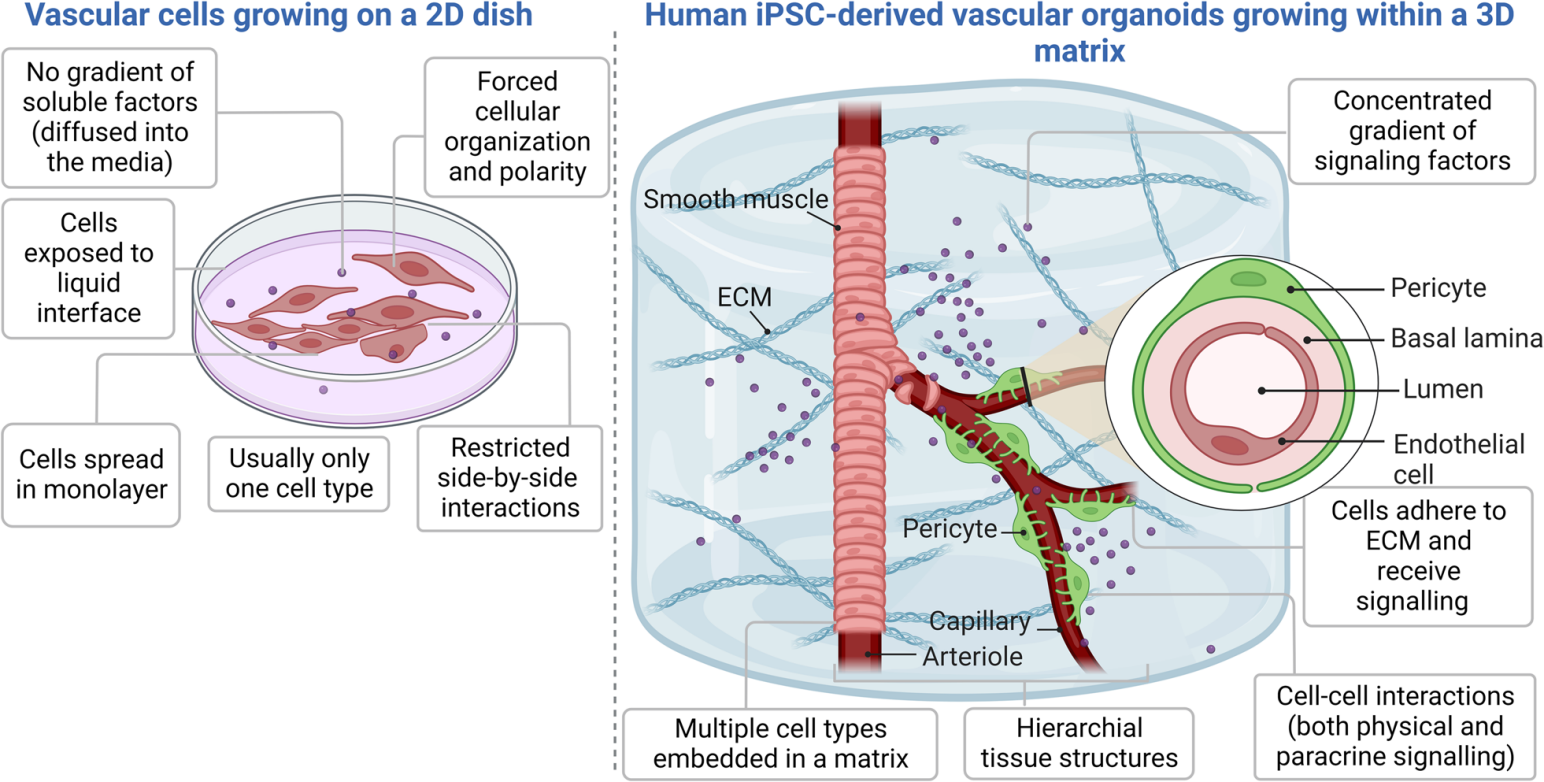
Advantages of Human iPSCs-Derived 3D Vascular Organoids Over Conventional 2D Models. A comparison between 2D (left) and 3D (right) systems highlights the advantages of human iPSCs-derived 3D vascular organoids. These patient-derived organoids closely resemble native blood vessels, containing crucial vascular cell types such as mural and endothelial cells, thus enabling better mimicry of native pathophysiological responses. Their personalized nature offers the potential for studying individualized drug responses, as hiPSCs-derived organoids retain patient-specific metabolic and hyperglycaemic memory from the original tissue/organ. These functional vascular organoids can be integrated with other organoids or engineered tissue constructs. The absence of irrelevant cells in the microenvironment and inclusion of all major vascular cell types streamline downstream experimental analyses. However, the simplified tissue complexity may be considered a limitation in constructing fully organotypic blood vessels

In comparison to traditional 2D cell cultures, 3D vascular organoids derived from induced pluripotent stem cells (iPSCs) offer numerous advantages, as illustrated in Fig. 1. While 2D cultures are simple and cost-effective, they have inherent limitations, including forced cellular organization, lack of signalling molecule gradients, and restricted physical cell contacts in two dimensions. Conversely, 3D organoid culture systems enable dynamic interactions between multiple cell types and the extracellular matrix (ECM), leading to a coordinated function and hierarchical organization within a biomimetic microenvironment [8–10].

One of the key advantages of iPSC-derived vascular organoids is their ability to better recapitulate native pathophysiological responses due to their multicellular population, which includes both mural cells and endothelial cells. These organoids facilitate reciprocal inductive interactions, allowing for the manifestation of intercellular dynamics and activity-dependent adaptations at a macro scale similar to native organs [11–15]. Additionally, these multicellular vascular structures contributes to their functionality as pivotal modules, enabling the assembly of vascular organoids with other organoids (such as brain, kidney, pancreas, intestine, and heart) to generate more complex and controllable vascularized tissues and organs [4, 10, 16–18]. Moreover, iPSC-derived vascular organoids hold great potential as personalized drug testing platforms because they retain epigenetic memory of the patient from which they are derived, making them more suitable for studying impaired vascular cell function leading to cardiovascular diseases [2, 19–23].

## Addressing current shortcomings of 3D organoids: emerging solutions

While 3D organoids offer immense potential, there are several challenges that need to be addressed. Table 1 highlights some of the most significant limitations of human organoids along with potential solutions.

**Table 1:** Current limitations of human organoids and suggested solutions to address drawbacks

	Limitations/challenges	Corresponding solutions/strategies
	Lack of microenvironment cells such as immune cells and stromal cells	Establishing co-culture of the organoids with microenvironment cells [6, 26]
	Often limited in size and developmental state, analogous to embryonic/ foetal organs rather than adult organs	Integration of other organoids with the VOs or ECs, to enhance size, complexity, oxygen/nutrient distribution, and adult-like paracrine signalling [4, 6, 45] Applying bioengineering methods and design principles to follow native organ multiscale structural orders and architecture [10, 16]
	Often heterogeneous and irreproducible structures at macroscale (i.e. 100’s of microns to millimetres) [29]	Implementing techniques to control spatial and temporal organoid formation, making it more deterministic [31] Developing differentiation protocols for region-specific organoids using microfluidics, matrix, or bioreactor approaches [30, 34]
	Spontaneous morphogenesis processes within cell aggregates with minimal to no exogenous control [29]	Making it more automated and continual in situ monitoring of organoid behaviours [32] and guiding pattern by environmental cue [31] Multisensory-integrated systems for automated and continual in situ monitoring of organoid behaviours [32]
	Lack of inter-organ communication, fundamentally mimicking a part of the human body, not the entire body	Multiple organoids connection by a chamber device, ‘organoid-on-a-chip’ technology [10, 16, 35]. Compartmentalization of distinct organoid types enables fine-tuned organoid–organoid communication, while preventing their uncontrolled fusion [32, 33]
	Variable cellular subpopulations, with unknown number and steps of intermediate cells particularly from hPSCs differentiation [37]	Developing a standardized differentiation protocol QC for presence of unwanted cell types by single-cell RNA-sequencing and other analyses Precisely controlling differentiation to desired cell types by blocking the formation of unwanted cell types or by overexpressing lineage-specifying transcription factors [37]
	Lack of a standardized protocol or guidelines to ensure quality and reproducibility	Establishing standardized protocol with collective efforts by multi-Centre scientific consortium Culturing under the well-defined culture media and ECM
	Uncertainty in the composition of matrix used for 3D organoids generation	Developing mechanically and chemically defined synthetic extracellular matrices for organoids culture [41–43]
	Low vascularization and maturity decrease organoid lifespan and increase necrotic core (nc) formation [4, 25]	Incorporating vascular organoids into other non-vascular organoids Long-term culture, vascularization with microfluidics, and animal transplantation [34]
	Relatively costly compared to 2d cultures, and fly, yeast, or worm models [8, 40]	Establishing widely accepted and used protocols for each type of organoid will decrease the cost progressively
	Difficulty in cryopreservation and recovery	Developing new cryopreservation protocols and devices adapted for multicellular tissue banking and long-term storage

One inherent issue with organoids is the absence of microenvironmental cells such as immune cells, stromal cells, and endothelial cells (ECs) [4, 24]. Co-culturing VOs with mesenchymal and/or immune cell populations has been proposed as a solution [4, 6, 25, 26]. However, the absence of a microenvironment in organoids can also offer advantages [8], emphasizing the importance of tailoring cellular complexity to suit specific studies. To increase the size and cellular complexity of organoids resembling mature organs, incorporating ECs for vascularization or assembling vascular organoids with other organoids has been suggested [4–6, 17, 18, 27]. These approaches help ensure sufficient oxygen and nutrient distribution, addressing a classic challenge in tissue engineering [28].

Generating large organoids (ranging from hundreds of microns to millimeters) introduces increased variability, hindering comparability between research groups [29, 30]. Adhering to design principles that follow the multiscale structural orders and architecture of adult organs can help address this issue [10, 16, 30]. To enhance reproducibility, Gjorevski et al. have proposed methodologies for deterministic organoid patterning, controlling spatial and temporal organoid formation rather than relying on a stochastic process [31]. Real-time sensors for monitoring biophysical and biochemical parameters can further promote reproducibility and performance [32]. Other proposed solutions include devising differentiation protocols for region-specific organoids, standardizing through microwell-based approaches, and implementing compartmentalization to enable fine-tuned organoid-organoid communication while preventing uncontrolled fusion [32–34]. Organoid-on-a-chip technologies and bioengineering methods facilitate multiple organoid connections [10, 16, 35].

Besides technical and bioengineering methodologies, part of the irreproducibility of organoid generation arise from variations in the differentiation of hPSCs towards a distinct type of organoid by organ-specific inducers (small molecules) [36]; resulting in heterogeneous cellular composition/subpopulations with an unknown number and steps of intermediate cells [37, 38]. Therefore, single-cell RNA-sequencing and other analyses are crucial for quality control to identify and eliminate unwanted cell types [8]. Precise control of differentiation into desired cell types can be achieved through strategies such as blocking the formation of unwanted cell types or overexpressing lineage-specifying transcription factors [37] as well as chemically defined media [37, 39]. Additionally, fluorescence-activated cell sorting (FACS) can selectively purify desired cell types, although its feasibility may vary depending on the differentiation process and the need for organoid disruption and cell dissociation.

The use of commercially available hydrogel matrices like Matrigel and collagen, derived from animal cells, in human 3D organoid generation introduces additional level of heterogeneity and irreproducibility [5, 40]. To address this, researchers are striving to develop mechanically and chemically defined synthetic extracellular matrices [41–43] for organoid culture. However, the presence of the ECM, essential for making a fully mature organoid structure, poses challenges during cryopreservation. It hinders sufficient and immediate infiltration of cryopreservation media, making efficient recovery and cell viability after freeze–thaw cycles more complex. Therefore, the development of cryopreservation protocols and devices specifically designed for large, multicellular organoid banking is crucial. Overcoming these challenges will ensure optimal cryopreservation outcomes and facilitate the widespread use of organoids in various research and clinical applications.

## Wide-ranging applications of human vascular organoids

**Fig. 2:**
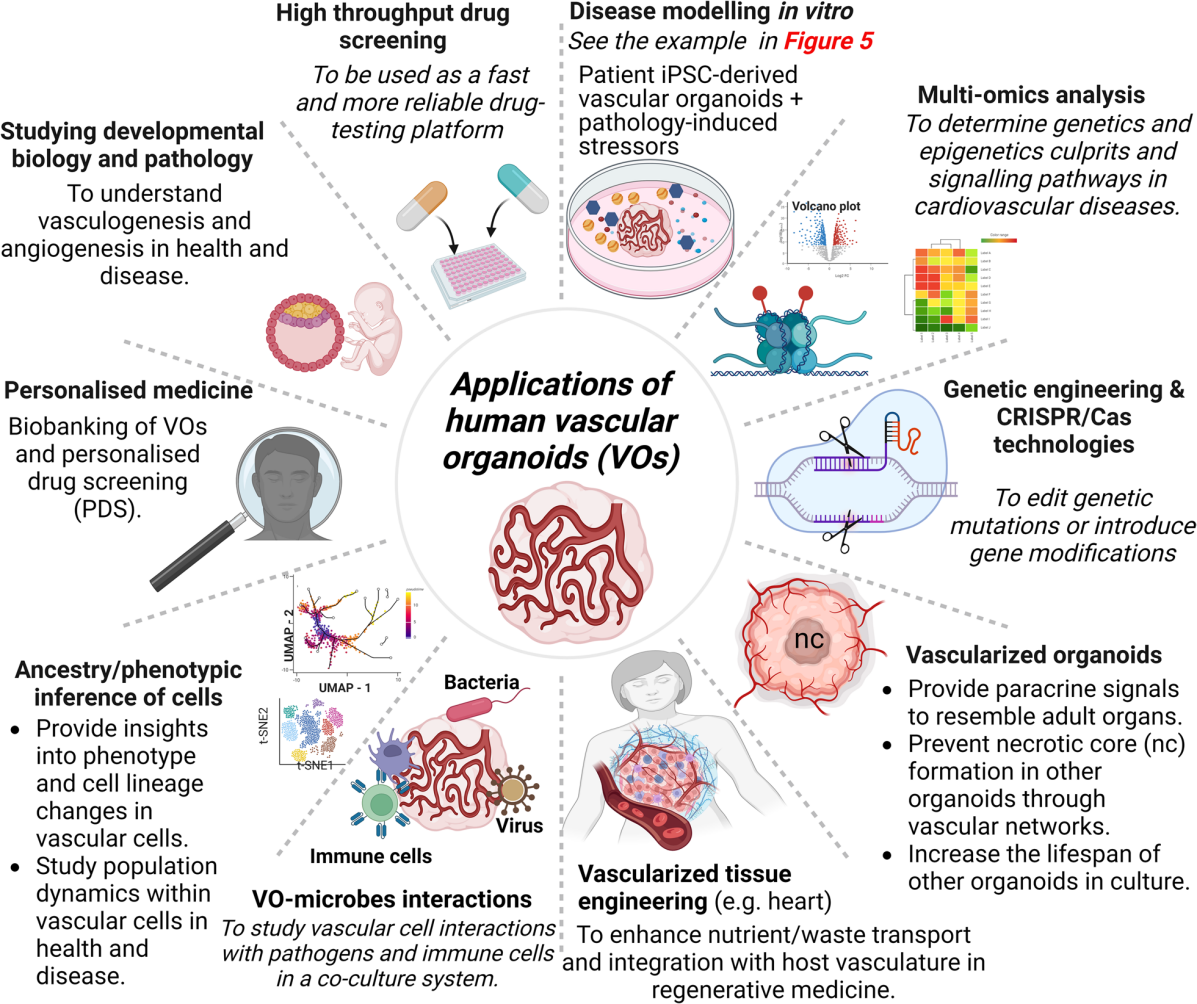
Potential Applications of Human Vascular Organoids. Human vascular organoids offer a wide range of potential applications, encompassing various fields such as basic biological and developmental science, disease modelling, drug screening, and regenerative medicine. These versatile organoids have the capacity to contribute to diverse research areas and hold promise for advancing our understanding of vascular biology and facilitating the development of novel therapeutic strategies

Human vascular organoids hold immense potential across various applications, as depicted in Fig. 2. One notable advantage of vascular organoids is their ability to integrate with other organoids, facilitating enhanced maturation and vascularization. By incorporating vascular cells, such as ECs and mural cells, vascular organoids contribute to the development and vascularization of other human organoids [4, 17, 18]. They also play a crucial role in tissue engineering, addressing challenges like necrotic core formation in large multicellular structures due to limited oxygen and nutrient supply [4, 27]. Moreover, vascular cells contribute to the maturation of other organoids through paracrine signaling, increasing their resemblance to adult organs [44, 45].

Organoids have also been instrumental in studying the interaction of blood vessels with pathogens and immune cell populations in a co-culture system [9, 46]. The outbreak of the COVID-19 pandemic in 2020 highlighted the critical role of organoids in research. Various organoid models, including those of the respiratory system, tonsils, intestines, heart, liver, brain, kidneys, eyes, pancreas, and vasculature, have been widely adopted to study SARS-CoV-2. These models have provided insights into the virus’s life cycle, cellular tropism, and pathogenesis [47–49]. For instance, studies have demonstrated the direct infection of human kidney and blood vessel organoids by SARS-CoV-2 and the use of soluble human ACE2 to block viral infection [50]. Another recent study has shown that stimulation of vascular organoids with SARS-CoV-2 antigens could increase pericyte uptake of SARS-CoV-2 or Spike glycoprotein and subsequently, through altering endothelial permeability and enhancing the risk of thrombotic complications, contribute to vasculopathy [51].

Organoids provide a promising platform for the establishment of living biobanks consisting of organoids derived from patients with diverse diseases. These biobanks serve as valuable resources for both basic and clinical research, as well as personalized medicine. In the context of vascular biology, vascular organoids derived from hPSCs offer a powerful tool to model and investigate the development of the vascular system in both healthy and diseased states. On the other hand, it is important to note that vascular organoids derived from adipose-derived stromal/stem cells are not suitable for developmental biology studies. However, these adipose-derived stromal/stem cell-derived vascular organoids have shown promise and find applications in other specific areas, which have been extensively reviewed elsewhere [40, 52].

iPSCs-derived vascular organoids (VOs) provide a valuable platform for investigating age-related cardiovascular diseases progression. While reprogramming can partially reset cellular aging, iPSCs retain an epigenetic memory and telomere length of the donor. By differentiating iPSCs into specific cell types affected by age-related conditions, like vascular organoids for cardiovascular diseases, researchers can analyse age-related alterations by comparing patient-derived iPSC-derived cells with healthy donor cells or cells from donors of varying ages [53]. Incorporating advanced aging models, such as extended culture or exposure to stressors, allows for the emulation of cellular aging and enhances the relevance of the model. iPSCs derived from donors with shorter telomeres might manifest aging-associated features due to telomere shortening during cell division. Importantly, while telomere length is one aspect of cellular aging, iPSCs derived from older donors with shorter telomeres may also exhibit other age-related epigenetic changes and functional shifts [54, 55].

Patient-derived organoids hold tremendous potential for predicting drug responses in personalized medicine [9, 56]. For instance, the response of stem cell-derived organoids to drugs used in cystic fibrosis treatment correlates with the patient’s response, making organoids an invaluable and cost-effective platform for drug testing [57–59]. Organoid assays, such as the intestinal swelling assay, serve as biomarkers for diagnostic decision-making in cystic fibrosis patients [40, 57, 60], allowing for personalized treatment strategies.

Vascular organoids can be used to explore the role of sex-based differences in various vascular diseases, such as cardiovascular diseases, stroke, and microvascular complications associated with diabetes. While strides have been taken in understanding sex-specific mechanisms in diabetic vascular issues [61], many questions persist, and evidence for sex disparities in microvascular disease is limited. Generating organoids from both male and female donors enables studying genetic influences on diseases. Integrating patient-derived data, such as genomic information and clinical profiles, with vascular organoid models can enhance the relevance and translatability of organoid-based findings. This will help to better understand disease etiology, predict disease progression, and personalize treatment strategies. Additional sex-specific factors, such as hormone levels that can impact disease susceptibility, progression, and response to therapies, can be added to the culture media of the VOs.

A significant advantage of organoids over animal models and clinical trials is their potential for large-scale in vitro amplification [8, 9]. This enables the generation of abundant materials for high-throughput drug screening, deep sequencing, single-cell technology, CRISPR screening, and gene/genome editing. The applications of organoids, including vascular organoids [2], are only in their early stages, and they hold great promise for unravelling disease aetiology, understanding human organ development, disease modelling, identifying novel therapeutic targets, and expanding treatment options.

## iPSCs’ potential to create functional blood vessels: exploring endothelial-mural cell interactions in vascular health and disease

**Fig. 3:**
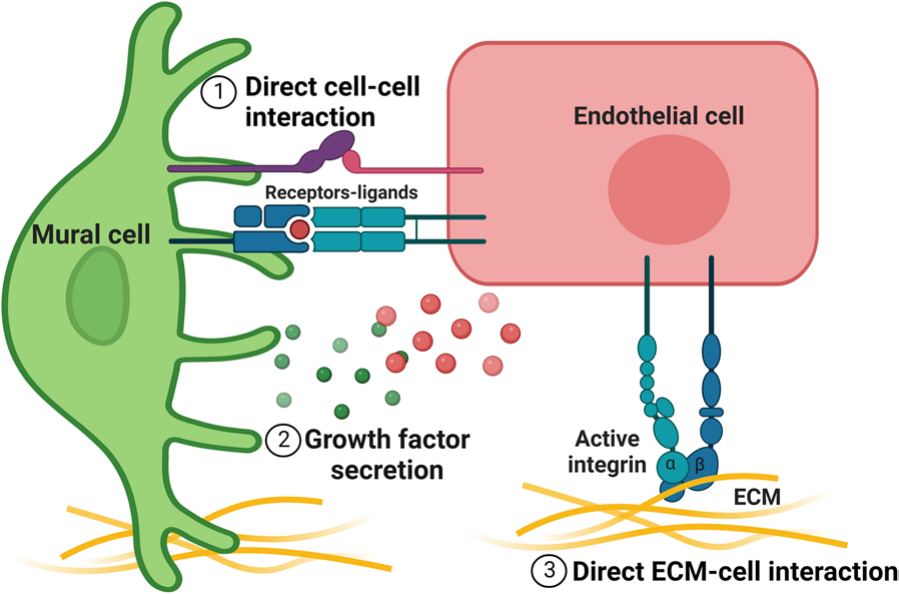
Importance of Interaction between Mural Cells and Endothelial Cells in Vascular Health. This figure highlights the significance of the interaction between mural cells and endothelial cells in ensuring proper blood vessel function. (1) Direct physical contact between mural cells and endothelial cells plays a crucial role in maintaining vascular integrity. (2) Both cell types communicate through the secretion of paracrine factors, further supporting the functional coordination of the blood vessel. (3) The extracellular matrix (ECM) also influences the behaviour and function of both cell types. Disruption of these intimate interactions can lead to the development of vascular diseases. Moreover, impairments affecting one cell type in vascular disease can have consequential effects on the other cell type. Mural cells exert a multifaceted impact on endothelial cells (ECs), regulating vasoconstriction and vasodilation, preserving vascular integrity and function, aiding vessel development and stabilization, and contributing to extracellular matrix (ECM) production for vasculature patterning. ECs, in turn, play crucial roles in lumen formation, secretion of vasoprotective factors, interaction with immune cells, and autocrine signalling. They also control mural cell recruitment, proliferation, migration, and specialization

iPSCs have the potential to generate fully functional blood vessels composed of vascular cells, including ECs and mural cells [2, 23]. The three main components of blood vessels are the basement membrane, ECs, and mural cells, which consist of vascular smooth muscle cells (vSMCs) and pericytes. ECs line the innermost vessel wall, and the mural cells closely cover endothelial cell tubes, even though heterogeneity exist within each cell type depending on the anatomical location and role [62]. Although ECs as primary building block of blood vessels can form tube-like structures independently, they are unable to form complex functional vasculature without support from mural cells and the ECM [63]. As illustrated in Fig. 3, both ECs and mural cells are essential components of blood vessels for proper function by reciprocal effects [2]. ECs are necessary for mural cell differentiation, specification, recruitment and attachment [64]. In turn, mural cells are required for normal vascular development, stabilization, maturation, homeostasis, remodelling, and specialized function in different organs [65, 66]. Therefore, the correct functioning of both ECs and mural cells is essential for a healthy endothelium and subsequently vascular health. Accordingly, impairment of one cell type due to cardiovascular risk factors will inevitably affect the other cell types and lead to vascular disease. For example, diabetes not only impairs the functions of ECs [67] and disturbs the communication between ECs and pericytes [68], but also affects mural cells. Alterations in mural cell density or abnormal interactions with the endothelium are implicated in several human pathological conditions including diabetic microangiopathy, retinopathy and nephropathy, venous malformation and hereditary stroke and lymphedema [68–70].

Previous studies on blood vessel pathophysiology have primarily focused on individual cell types or co-culture systems of ECs and mural cells in 2D culture or in vivo experiments with confounding factors. However, to fully understand vascular development and impairment in cardiovascular diseases, it is necessary to analyse organotypic blood vessels. Signalling molecules secreted from mural cells play a crucial role in the formation of organotypic vessels, and direct cell–cell contacts and secreted growth factors facilitate communication between ECs and mural cells [71, 72]. Creating in vitro organotypic models that include both cell types as well as the ECM is essential to unravel the complexities of endothelial-mural cell signalling. Such information about the mediators of interactions between vascular cells during blood vessel formation is technically difficult to discern from in vivo studies [63].

**Fig. 4:**
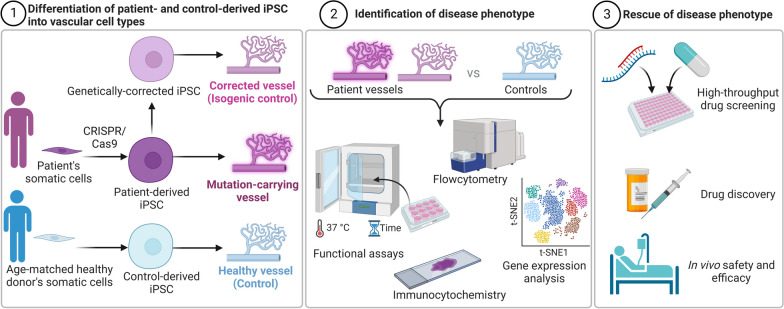
Utilizing Patient-Derived Induced Pluripotent Stem Cells (iPSCs) for In Vitro Disease Modelling of Vascular Complications. The figure showcases the application of patient-derived iPSCs in the in vitro modelling of vascular complications. (1) Patient-derived iPSCs are differentiated into blood vessel organoids comprising all vascular cell types. CRISPR-Cas9 technology allows the generation of isogenic iPSC control lines by repairing mutations or introducing patient-specific gene editions or other genes of interest. (2) By comparing these intact blood vessels from individuals with vascular diseases to healthy controls, researchers can identify the cellular and molecular mechanisms underlying the disease phenotype. (3) These vascular organoids serve as valuable tools for in vitro disease modelling of specific vascular complications and can be utilized in high-throughput drug screening to discover novel therapeutic agents, facilitating pre-clinical and/or clinical trials

To enhance our understanding of endothelial-mural cell signaling and to develop effective in vitro models for studying vascular health and disease, it is challenging to directly obtain both ECs and mural cells from patients and expand them in unlimited quantities for in vitro studies, including drug screening. Alternatively, due to the differentiation capabilities of stem cells, in particular iPSCs, reprogramming technologies have offered strategies to generate patient-specific disease models [7, 73]. The unique characteristics of iPSCs have allowed for the development of protocols to effectively reprogram iPSCs into both functional ECs and mural cells [2], enabling the improved understanding of specific roles of certain cell types within the vasculature in health and disease (Fig. 4). We have previously shown that iPSCs-derived ECs from non-diabetic individuals can sprout normal blood vessels, however, the same does not hold for those from diabetic patients [22, 74, 75]. We have shown that there is a unique signature of vascular dysfunction in diabetic iPS-ECs [75]. Diabetic iPSCs seem to retain metabolic memory [20] or hyperglycemic memory [19] epigenetically, and as they differentiate into blood vessels, they become inflamed and are more prone to oxidative stress leading back to CVDs [21]. Studies have also shown that transcriptional memory allows certain disease-related genes to respond more strongly to previously experienced signals/condition, resulting in faster and greater signal-dependent transcription of these genes [76, 77]. The previously experienced condition in the case of diabetes is a hyperglycaemic and pro-inflammatory environment. It is therefore best to simulate diabetic conditions by risk factors/inducers such as applying high glucose concentration and/or inflammatory factors such as TNF-a and IL-6 [23], to instigate pathological pathways in diabetic-patient iPSCs-derived vascular cells. These vascular organoids have been used to investigate vascular complications in diabetes, such as basement membrane thickening, by culturing them in a high-glucose medium with pro-inflammatory cytokines. Additionally, the organoids can be transplanted into diabetic mice to create chimeric humanized mouse models, allowing the modelling of diabetic vasculopathy in vivo. These models accurately recapitulate diabetic vascular features, including basement membrane thickening, narrowed lumen, and vessel regression. They also provide an opportunity to assess functional vessel parameters, such as permeability and blood flow, as well as perform preclinical toxicology testing.

Studies have also used coculture techniques combining or bioprinting either iPSC-derived cell types or differentiated primary cells to generate better functional vascular networks for their investigation [78–80]. However, many of these co-culture systems are still considered as a 2D culture system. Patient iPSCs-derived vascular organoids are the latest innovation for research and are being used as the quintessential model for both in vivo and in vitro studies. In particular, the presence of both mural cells and ECs in the vascular organoids reveals new data that was previously unattainable. This information may prove vital in the fight against diabetes and cardiovascular diseases. Furthermore, studies have revealed the importance of paracrine signalling in controlling cellular heterogeneity and highlighted that multicellular populations such as organoids better mimic native pathophysiological responses [11].

## Strategy for generating 3D disease models of diabetic vasculopathy in vitro

**Fig. 5:**
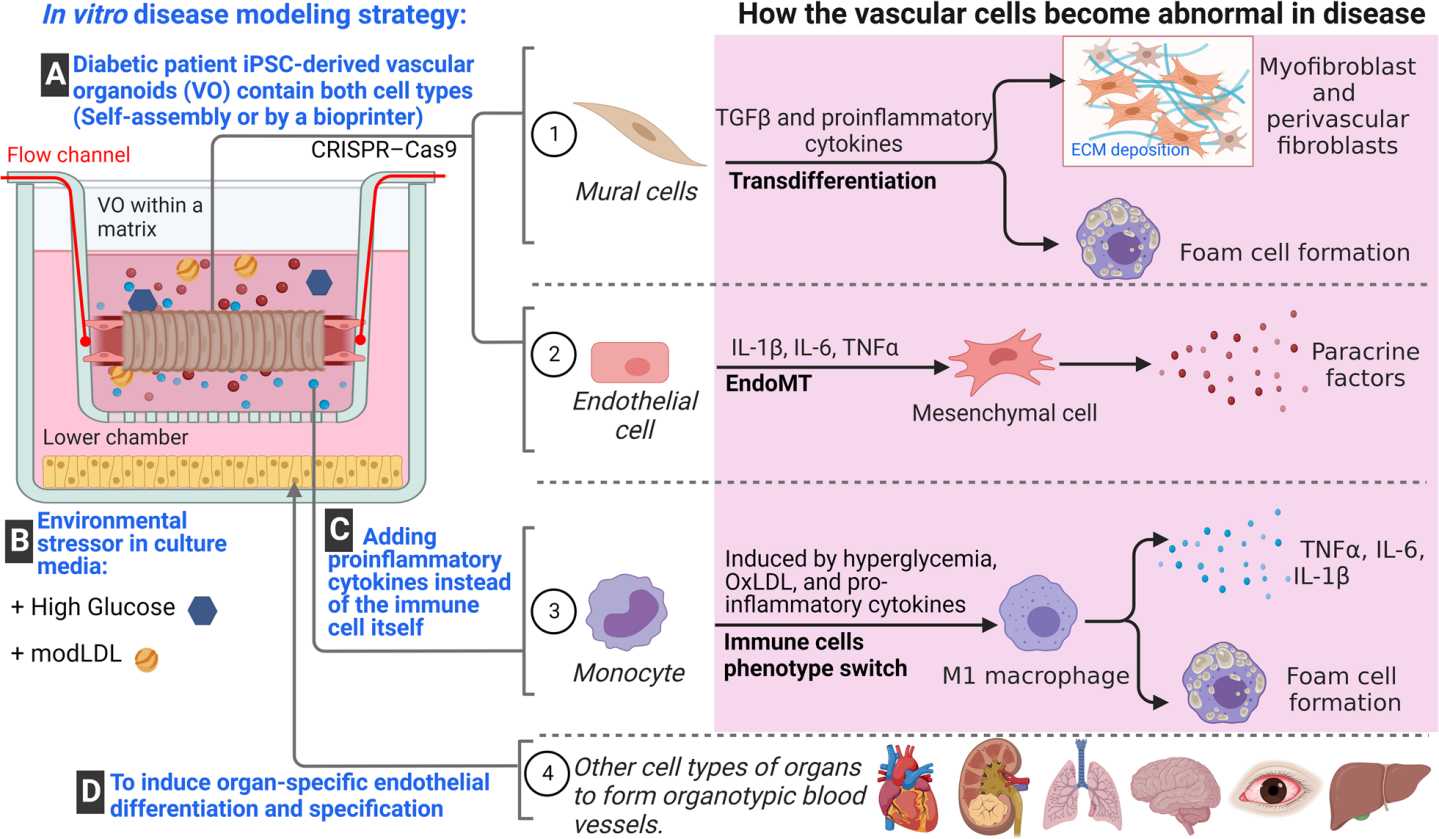
A Strategy for In Vitro Modelling of Vascular Complications: Focus on Diabetic Vasculopathy/Angiopathy. The right panel highlights the requirement of four different cell types for comprehensive modelling of vascular complications. The blue texts (**A–D**) indicate how each cellular and environmental element is incorporated to generate an intact organoid model of diabetic vasculopathy/angiopathy, elucidating the mechanisms underlying abnormal vascular cell behaviours (pink box). **A** illustrates the generation of blood vessel organoids from patient-derived induced pluripotent stem cells (iPSCs), enabling the study of mural cell transformation, endothelial mesenchymal transition (EndoMT), and genetic modifications. Also, microfluidic technology allows the introduction of different flow types and facilitates the formation of perfused blood vessel organoids. **B** demonstrates the simulation of environmental stressors by adding pathological risk factors to the culture media. **C** shows the incorporation of immune cells in a co-culture fashion or through the addition of pro-inflammatory cytokines. **D** addresses the ongoing challenge of including organ-specific cell types in the model to induce organ-specific vascular phenotype. EndoMT; endothelial mesenchymal transition

To develop in vitro disease models of diabetic vasculopathy, we can draw upon the pathophysiological events that occur during the initiation and progression of the disease. In Fig. 5, we present possible strategies for modelling diabetic vasculopathy/angiopathy using hPSC-derived vascular organoids.

The onset and progression of diabetic vasculopathy is heavily associated with the alteration and dysfunction of ECs and smooth muscle cells, which are accompanied by vascular inflammation and fibrosis [81]. Through either glucose, low-density lipoprotein (LDL), epigenetic modifications, or a combination of all, monocytes are activated and differentiated into a more inflammatory cell lineage. This activation triggers the release and high expression levels of pro-inflammatory cytokines, including TNF-α, IL-1β, and IL-6, most likely through the activation of NF-κB, and toll-like receptors (TLR2 and TLR4) [82]. Exposure of ECs to such factors triggers ECs to express adhesion molecules such as VCAM1, E-selectin, and ICAM1, as well as inflammatory cytokines including monocyte chemotactic protein and IL-1β [83]. This consequently creates an environment that encourages the recruitment of circulating monocytes and their subsequent differentiation into macrophages, which then internalize oxidised LDLs to become foam cells. Both macrophages and foam cells thereon have central roles in atherosclerotic plaque development, local inflammation, and the promotion of thrombosis, contributing to vascular disease development. Moreover, this pro-inflammatory environment, particularly the expression of TGFβ, is known to stimulate endothelial to mesenchymal transition (EndoMT), a process in which endothelial cell function lessens and diminishes, affecting for example cell to cell junctions, allowing for increased permeability, facilitating leukocyte trafficking, basement membrane degradation and cell migration [84]. It has also been reported that EndoMT is directly linked to the immune response upon exposure to proinflammatory stimuli such as TNF-α, IL-6, IL-1β, high glucose, or oxidised LDL [85]. The phenotypic modulation in which ECs suppress constituent endothelial properties gives rise to mesenchymal fibrotic cells. Similarly, in response to the inflammatory cues, mural cells also transdifferentiate [65, 86]. This fibrotic shift results in the generation of reactive oxygen species, stimulate neurohumoral responses, activates growth factor cascades (TGF-β/Smad3 and PDGFs), induces pro-inflammatory cytokines and chemokines, generates advanced glycation end-products (AGEs) and stimulates the AGE-RAGE axis, as well as causes the upregulation of fibrogenic matricellular proteins, all of which contribute to the onset and progression of diabetic vasculopathy [87]. As such, to effectively recapitulate diabetic vasculopathy in vitro, these three cellular components contributing to vascular dysfunction, which have been summarised in Fig. 5, need to be presented in a disease model.

To accurately recapitulate diabetic vasculopathy in vitro, it is crucial to include these three cellular components (ECs, SMCs, and immune cells) contributing to vascular dysfunction in the disease model. Vascular organoids derived from patient-specific pluripotent stem cells can replicate the multicellular repertoire of human physiology in vitro. These organoids, when exposed to disease-specific stimuli, such as high glucose and LDL, can maintain a metabolic and hyperglycemic memory of the disease state. In addition, to mimic the immune response observed in diabetes, it is essential to incorporate inflammatory components, either through the direct addition of pro-inflammatory cytokines or the co-culture of monocytes that can secrete the appropriate inflammatory cytokines. The combination of cells with epigenetic memory, environmental factors, and inflammatory cues might accurately reflects the pathophysiology of the diabetic vasculature. Studies have successfully differentiated pluripotent stem cells into vascular organoids that contain capillary networks of ECs, pericytes, and SMCs, which were exposed to a diabetogenic medium to emulate the hyperglycemic and inflammatory microenvironment found in diabetes [3]. These diabetic organoids displayed thicker vascular basement membranes and reduced capillary density compared to non-diabetic controls.

While iPSC-derived vascular cells can self-organize into mature vasculature, an alternative approach is to bioprint various cell sources together. Bioprinting studies using patient-derived cells have successfully mimicked vasculature in vitro. For example, a 3D vascular model was developed using in-bath coaxial cell printing, encompassing the geometrical and cellular configurations of mature vessels [88]. The system contained connective tissue, smooth muscle, and an endothelium. To study dysfunction, they added a high concentration of LDL into the system and monitored the onset of inflammation through an increase in ICAM-1 expression, as well as introduced immune cells to mimic the key features of atherosclerosis. The efficacy of the model to recapitulate human vasculature was confirmed by atorvastatin treatment, a typically prescribed medication to diabetic patients to reduce LDL levels, which they reported to decrease the number of foam cells. Similarly, to study diabetic nephropathy, intending to identify anti-diabetic drug responses, another group effectively used a combination of patient-derived cells and bioprinting to create a system in which two separate tubular structures, one with proximal tubule ECs and the other with glomerular microvascular ECs were positioned adjacently to create a mature vascular 3D model with a high glucose reabsorption efficiency due to the active crosstalk [89]. Although mural cells were missing in this study, to further mimic the diabetic environment, the system was exposed to high glucose levels. Subsequent analysis revealed the cells to effectively mimic diabetic vasculature, exampled through damaged cell junctions and high levels of oxidative stress. An advantage to such bioprinted methods compared to vascular-derived self-assembling organoids is the incorporation of perfusion which mimics blood flow and thus creates a more representative vascular disease model.

Currently, blood vessel organoid with perfusion ability is only available after transplantation in vivo [3]. Therefore, further improvement to the proposed model (Fig. 5) is using microfluidic systems to establish perfused blood vessel organoids in vitro, allowing to investigate functional parameters including shear stress, permeability/vascular leakage, defective vascular organization, or adhesion/extravasation of immune cells and microbes, among others [10, 16, 35, 73, 90, 91].

## Generating organ-specific blood vessels In Vitro

Blood vessels in different organs exhibit significant variations in terms of metabolism, transcriptional profile, morphology, structure, and function [92, 93]. As a result, organs respond differently to various risk factors, including diabetes. To effectively combat organ-specific vascular complications and gain a better understanding of the underlying cellular and molecular mechanisms, it is crucial to develop in vitro models of blood vessels that incorporate organ-specific features.

While various 3D strategies can be used for modelling vascular complications like diabetic vasculopathy, a shared feature among successful models is the combination of patient-derived cells with an epigenetic memory and disease-specific cues. However, a persisting challenge to the proposed model is the derivation of organotypic blood vessels that recapitulate the biological complexity of native tissues, since vascular cells are phenotypically and functionally different for each organ [72, 94–97]. Therefore, including organ-specific cell type in the lower chamber of the proposed model might induce the formation of organotypic blood vessel to some extent. Alternatively, organotypic growth factors can be applied into the culture media. Other key elements to adapt organ-specific vessel type, size, shape, and function are environmental signals such as mechanical forces and cell–matrix interactions that most likely achievable by using organ-specific hydrogels with spatiotemporal tuneable stiffness [98–100].

Additionally, research have revealed that blood vessels possess a memory of their cellular origins. The identity of vascular cells within a vessel is not solely determined by signals from the surrounding tissue but also by the identity of their parental cells, known as cells of origin [55, 101–105]. Therefore, the tissue source of cells used to generate iPSC lines also plays a role, as residual epigenetic memory can influence the phenotype of iPSCs and bias their differentiation towards a specific lineage [55, 104, 106]. Human iPSCs have been derived from various cell sources, including fibroblasts [7], keratinocytes, and mononuclear cells from healthy individuals or patients [22, 107, 108]. These iPSCs can then be differentiated into specific lineages, including ECs and mural cells [2, 75]. However, to fully recapitulate the structure and function of the blood vessels, ECs or endothelial progenitor cells (EPCs) are considered ideal cell sources due to their epigenetic memory for blood vessel differentiation. Two potential sources are tissue residential ECs/EPCs or Colony Forming Units-Endothelial Progenitor Cells (CFU-EPCs) of adult’s peripheral blood and umbilical cord blood. The latter is a readily available source with several advantages than tissue residential ECs/EPCs, including no need for enzymatic digestion to isolate and culture, more efficient reprogramming potential, and cost-effectiveness for clinical translation [109, 110].

While generating iPSCs from ECs/EPCs has been achieved [109, 111], the molecular basis and mechanisms of retained epigenetic memory are not fully understood. Further investigation and ongoing efforts are needed to determine if these strategies successfully replicate organ-specific features of blood vessels.

## Conclusion and future perspectives

Human iPSCs hold great promise for generating functional blood vessels composed of ECs and mural cells. The development of in vitro organotypic models that incorporate both cell types and the ECM is crucial for understanding vascular development and disease mechanisms. Patient iPSC-derived vascular organoids are a valuable tool for studying diabetes and cardiovascular diseases, providing insights into the complex interactions between different cell types within the vasculature. These organoids offer opportunities for both in vitro and in vivo investigations, paving the way for improved disease modelling and therapeutic development.

In this review, we have demonstrated that human PSCs-derived 3D organoids are superior in some features/applications and complementary to conventional 2D cell culture methods, animal models, and clinical trials. As an example, we showed how hPSCs-derived vascular organoids can recapitulate the in vivo architecture, geometric features, functionality, and epi/genetic signature of the original patient. These VOs can be used for modelling complex hereditary and non-hereditary, and/or infectious diseases in vitro.

In addition to discussing breakthroughs, challenges, and potential solutions in the field of organoids, we have emphasized the critical role of multicellular interactions and environmental cues in successfully modelling diabetic vasculopathy or angiopathy in vitro using vascular organoids as a platform. This blueprint can be applied to study other vascular complications in cardiovascular diseases.

Given the current challenges in the field of organoids, the convergence of stem cell technologies and bioengineering methods, such as microfluidics, nanofluidics, automation systems, and adjustable matrices, is essential. This convergence necessitates increased multi- and interdisciplinary collaborations to drive further improvements. With the help of various bioengineering tools, including microfluidics, microwells, Transwell systems, matrices with adjustable stiffness, and bioreactors, reproducibility can be enhanced, leading to the development of highly mature, dynamic, and organotypic blood vessel organoids in the near future.

The knowledge and information presented in this article will guide the scientific community in generating robust and powerful human PSC-derived vascular organoids for disease modelling of various vascular complications, including diabetic vasculopathy or angiopathy, in an organ-specific manner. The model described here can serve as a blueprint for in vitro disease modelling of other types of organoids by considering disease-specific stressors (environmental inducers or risk factors) and selecting the appropriate source of stem cells or differentiated cells (or a combination thereof) to obtain the desired cellular components. These disease models in vitro offer unprecedented opportunities for applications in basic and developmental biology, drug screening, and personalized translational medicine, ultimately advancing human health.

## Data Availability

Not applicable.

## Abbreviations

3D: Three dimensional
iPSCs: Induced pluripotent stem cells
hPSCs: Human pluripotent stem cells
VOs: Vascular organoids
ECM: Extracellular matrix
ECs: Endothelial cells
SARS-CoV-2: Severe acute respiratory syndrome coronavirus 2
ACE2: Angiotensin converting enzyme 2
CRISPR: Clustered regularly interspaced short palindromic repeats
vSMCs: Vascular smooth muscle cells
CVD: Cardiovascular diseases
EndoMT: Endothelial to mesenchymal transition
AGEs: Advanced glycation end-products
CFU-EPCs: Colony forming units-endothelial progenitor cells
